# Host predilection and transmissibility of vesicular stomatitis New Jersey virus strains in domestic cattle (*Bos taurus*) and swine (*Sus scrofa*)

**DOI:** 10.1186/1746-6148-8-183

**Published:** 2012-10-03

**Authors:** Paul F Smith, Elizabeth W Howerth, Deborah Carter, Elmer W Gray, Raymond Noblet, Roy D Berghaus, David E Stallknecht, Daniel G Mead

**Affiliations:** 1 Department of Entomology, College of Agriculture and Environmental Sciences, University of Georgia, 120 Cedar Street, 413 Biological Sciences Building, Athens, GA, 30602, USA; 2 Department of Pathology, College of Veterinary Medicine, University of Georgia, 501 D.W. Brooks Drive, Athens, GA, 30602, USA; 3 Department of Population Health, College of Veterinary Medicine, University of Georgia, 953 College Station Road, Athens, GA, 30605, USA; 4 Southeastern Cooperative Wildlife Disease Study, College of Veterinary Medicine, University of Georgia, 589 D.W. Brooks Drive, Wildlife Health Building, Athens, GA, 30602, USA

**Keywords:** Contact transmission, Insect transmission, Host predilection, Vesicular stomatitis virus

## Abstract

**Background:**

Epidemiologic data collected during epidemics in the western United States combined with limited experimental studies involving swine and cattle suggest that host predilection of epidemic vesicular stomatitis New Jersey virus (VSNJV) strains results in variations in clinical response, extent and duration of virus shedding and transmissibility following infection in different hosts. Laboratory challenge of livestock with heterologous VSNJV strains to investigate potential viral predilections for these hosts has not been thoroughly investigated. In separate trials, homologous VSNJV strains (NJ82COB and NJ82AZB), and heterologous strains (NJ06WYE and NJOSF [Ossabaw Island, sand fly]) were inoculated into cattle via infected black fly bite. NJ82AZB and NJ06WYE were similarly inoculated into swine.

**Results:**

Clinical scores among viruses infecting cattle were significantly different and indicated that infection with a homologous virus resulted in more severe clinical presentation and greater extent and duration of viral shedding. No differences in clinical severity or extent and duration of viral shedding were detected in swine.

**Conclusions:**

Differences in clinical presentation and extent and duration of viral shedding may have direct impacts on viral spread during epidemics. Viral transmission via animal-to-animal contact and insect vectored transmission are likely to occur at higher rates when affected animals are presenting severe clinical signs and shedding high concentrations of virus. More virulent viral strains resulting in more severe disease in livestock hosts are expected to spread more rapidly and greater distances during epidemics than those causing mild or inapparent signs.

## Background

Most vesicular stomatitis viruses have broad host ranges, infecting a large number of vertebrate and insect species [1]. In the United States, vesicular stomatitis New Jersey virus (VSNJV) is one of the causative agents of vesicular stomatitis (VS) in domestic livestock. Clinically affected animals typically present with vesicular lesions on the muzzle, tongue, lips, or coronary band, and occasionally the teats [2-4]. Vesicles usually rupture within 24-48 hr, leaving reddish ulcerations, which begin healing in 6-7 d [3]. Excessive salivation and a loss of appetite can occur when lesions are around the muzzle or in the oral cavity. In experimental settings, virus transmission by various biologically relevant routes including biological [5-9] and mechanical [10] insect transmission and animal-to-animal contact [11-14] has been demonstrated.

Vesicular stomatitis has been described as sporadically epidemic in the western United States [15], with outbreaks occurring in 1982-83, 1984, 1995, 2004-2006, and most recently in 2009. Horses and cattle are the primary livestock hosts affected during outbreaks, although swine can be affected as well. During the 1982-83 VSNJV epidemic, VSNJV infected livestock were confirmed in 14 states. Of the 614 animals confirmed to be infected during this epidemic, 68% were cattle [16]. In contrast, during the 1995 VSNJV epidemic in which clinically affected livestock were identified in six states, 71% of the foreign animal disease investigations and 78% of the positive premises involved horses [17]. During the 2004-2006 VSNJV epidemic, horses accounted for 78% of the 1,283 animals confirmed to be infected with VSNJV [18]. VSNJV infected animals were reported in nine states.

Phylogenetic analyses of VSNJV isolates obtained during these epidemics demonstrate that the virus lineage associated with each epidemic is distinct from that associated with a different epidemic (i.e. each outbreak is associated with a different VSNJV strain) [19,20]. When this is examined in conjunction with epidemiologic data collected during outbreaks, it appears that individual VSNJV strains have distinct host predilections. This theory is supported by reports of previous unsuccessful attempts to produce VS in cattle by experimental inoculation with swine and equine VSNJV strains [21,22], and by a report of decreased virulence of a 1982 bovine VSNJV strain in experimentally infected pigs [23]. If real, such host predilections could have a profound impact on the current understanding of transmission, both animal-to-animal and vector-borne, as well as the overall epidemiology of VSNJV.

Laboratory challenge of domestic livestock with homologous and heterologous VSNJV strains transmitted through black fly bite to investigate potential viral predilections has not been thoroughly investigated. Here, our objective was to more closely evaluate VSNJV host predilection in cattle experimentally infected with homologous and heterologous VSNJV strains and in swine infected with heterologous VSNJV strains. Swine have previously been infected with homologous strains [12] and this was not repeated in the current study. Differences in clinical presentation and extent and duration of viral shedding were monitored. Contact transmission in cattle was evaluated because this is thought to have been the route responsible for continued livestock infection during the winter of the 1982–83 epidemic [16].

## Results

### Host predilection

#### Cattle

**Figure 1:**
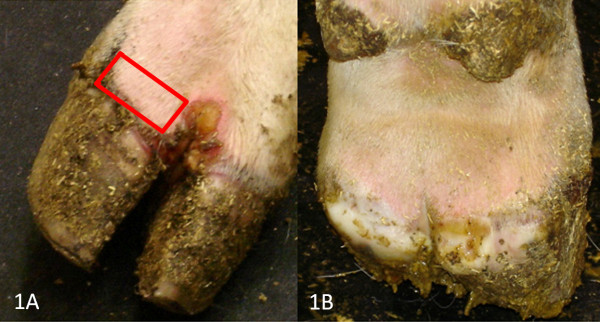
**Coronary band of steer bitten by black flies infected with NJ82AZB.** A. Anterior view showing fly feeding site (outlined in red) and secondary lesion. B. Posterior view showing development of severe secondary lesion around the entire circumference of the same coronary band.

Clinical disease (formation of vesicular lesions, virus shedding, and seroconversion) was detected when feeding occurred on the lower lip or coronary band for all viruses tested (Table 1). Virus was detected from neck bite site swabs of the NJ82AZB and NJ06WYE neck inoculated (group 3) animals at 24 hr post-infection; vesicular lesions were not present on group 3 animals. The highest viral swab titer detected on an animal bitten on the lower lip by black flies infected with NJ82AZB (Table 1) was 10^6.1^ TCID_50_/ml. The highest maximum swab titer observed from animals infected with either NJ82COB or NJOSF was 10^4.8^ TCID_50_/ml. There was no significant difference between viruses with respect to maximum viral titer or duration of shedding, although the estimated mean duration of shedding was 2-3 days longer for NJ82AZB than for any other strain (Table 2). Cattle infected with NJ82AZB also had the highest mean clinical score, which was significantly greater than that of cattle infected with NJOSF. The positive control animal in the NJ82AZB group failed to become infected via scarification of the lip or coronary band, and did not seroconvert against the virus. Lameness and behavioral changes were only observed in animals inoculated with NJ82COB and NJ82AZB. Steers bitten on the coronary band by black flies infected with NJ82AZB developed severe secondary lesions that spread from the initial inoculation site on the lateral claw, across the inter-digital space and around the entire circumference of the hoof (Figure 1A and 1B). These animals would remain lying down unless forced to rise, and when standing would avoid bearing weight on the affected hoof and limp severely when moving. Two of the three animals inoculated on the lower lip with this virus developed large secondary lesions on the tongue, which in one case resulted in sloughing of the entire epithelial layer, leaving a raw, reddened surface, which had begun healing by PID 12. Steers inoculated on the lower lip with NJ82COB similarly developed secondary lesions on the tongue and exhibited obvious care when feeding, although a decrease in feed consumption was not observed. Obvious physical discomfort or lameness was not observed for any animals infected with either NJ06WYE or NJOSF.

**Table 1:** Individual clinical outcomes for cattle infected with various strains of VSNJV

**Virus**	**Animal ID**	**Inoculation site (No. flies feeding)**	**Days of viral shedding (PID)**	**Max virus titer log**_**10**_**TCID**_**50**_**/ml (Location)**	**SN titer**
NJ82AZB	31	Lip (6)	1,4-6	5.26 (Lip)	>256
	36	Lip (14)	1,3-4,6-8	4.17 (Lip)	>256
	235	Lip (8)	1-7	6.1 (Lip)	>256
	236	CB (3)	2, 4-5	3.1 (CB)	>256
	238	CB (3)	2,4-8	2.26 (Tonsil)	>256
	32	Neck (12)	1	3.26 (Neck)	64
	33	Neck (8)	1-2	3.8 (Neck)	32
	39	CB/Lip (Control)	-	-	<4
NJ82COB	3	Lip (5)	1	2.17 (Lip)	>256
	26	Lip (1)	3-7	3.05 (Oral)	>256
	30	Lip (6)	-	-	16
	35	CB (6)	7	2.60 (CB)	>256
	27	CB (3)	4,7	3.10 (CB)	>256
	28	Neck (10)	-	-	8
	29	Neck (2)	-	-	<4
	34	CB/Lip (Control)	1, 4-7	3.05 (Lip)	64
NJ06WYE	137	Lip (10)	1, 4-5	4.05 (Lip)	128
	278	Lip (7)	-	-	64
	525	Lip (4)	1-7	6.05 (Lip)	>256
	443	CB (6)	2-3	5.05 (CB)	>256
	566	CB (0)^a^	2-4	4.17 (CB)	>256
	450	Neck (6)	1	2.80 (Neck)	32
	508	Neck (4)	1	2.17(Neck)	<4
	420	CB/Lip (Control)	1-2, 5	2.39 (Lip)	128
NJ OSF	40	Lip (6)	4-5	2.26	64
	41	Lip (7)	2, 4-6	2.8 (Oral)	128
	43	Lip (10)	1-6	4.8 (Lip)	>256
	38	CB (2)	-	-	<4
	NT	CB (4)	5	2.39 (CB)	64
	42	Neck (2)	-	-	64
	44	Neck (3)	-	-	64
	45	CB/Lip (Control)	1-2, 4-5	3.8 (Oral)	>256

Coronary band (CB) is abbreviated throughout the table.

^a^Infected black flies were observed biting the coronary band of animal 566, however blood was not detected when flies were dissected.

**Table 2:** Mean viral shedding days, maximum viral titers, and clinical scores by virus type in cattle bitten by black flies infected with different VSNJV strains

	**Virus**				***P***
	**NJ82AZB**	**NJ82COB**	**NJ06WYE**	**NJOSF**	
Viral Shedding Days	5.2	2.1	3.0	2.6	0.103
Maximum Viral Titer (log_10_ TCID_50_/mL)	5.46	2.77	5.36	4.1	0.132
Clinical Scores					
Lesion	2.8	1.4	1.4	1.0	0.041
Shedding	1.0	0.2	0.6	0.6	
Lameness	0.6	0.2	0.0	0.0	
Total	4.4^a^	1.8^a,b^	2.0^a,b^	1.6^b^	

Means with a superscript in common do not differ at the 5% level of significance.

*n= 5 cows/group.

#### Swine

Clinical disease was detected in all swine infected on the planum rostrale (snout) for both viruses tested. These animals developed large, vesicular lesions that coalesced to cover the entire planum surface, receiving the maximum clinical scores of 5. Extent and duration of viral shedding were similar between the two viral strains (Table 3). One animal infected on the coronary band with NJ82AZB developed a vesicle on the coronary band and VSNJV was isolated from both the coronary band and the mouth of this animal. This animal would limp noticeably when moving around in its enclosure. The animal infected on the abdomen did not develop any clinical signs of disease, but seroconversion was detected (Table 3).

**Table 3:** Clinical and serological response of domestic swine after exposure to VSNJV infected black flies

**Virus**	**Animal ID (Inoculation site)**	**Days of viral detection (PID)**	**Max viral tite log**_**10**_**TCID**_**50**_**/ml [location]**	**SN titer (PID)**
NJ82AZB	42 (CB)	-		<4 (11)
	50 (CB)	4-5	2.79 [CB]	>256 (11)
	55 (abdomen)	-		64 (11)
	39 (snout)	1-5	6.33 [nasal]	>256 (11)
	45 (snout)	1-5	6.5 [nasal]	>256 (11)
	49 (snout)	3-5	4.45 [oral]	>256 (11)
NJ06WYE	43 (snout)	1-4	6.0 [nasal]	32 (6)
	48 (snout)	2-5	6.5 [nasal]	64 (6)
	52 (snout)	1-4	6.33 [nasal]	32 (6)

Coronary band (CB) is abbreviated throughout the table.

### Animal-to-animal contact

In the initial contact-transmission experiment (Table 4 Experiment 1), primary animals inoculated by the bite of infected black flies with NJ82AZB developed lesions on the lip and VSNJV was detected on PID 4, and 7. Maximum viral titers from swabs of each of these two animals were 10^5.26^ TCID_50_/ml and 10^4.05^ TCID_50_/ml, respectively from lip swabs of both animals on PID 4. Positive swab samples were not recovered from any of the contact animals for this virus, and all animals remained seronegative throughout the experiment. One primary animal infected with the NJ95COE yielded a maximum virus titer of 10^3.05^ TCID_50_/ml on PID 4, and positive swab samples were collected on PID 4 and 7. Virus was not collected from swab samples of the second primary animal. Again, no positive VSNJV isolates were detected from any of the contact animals, and all remained seronegative throughout the experiment (Table 4).

**Table 4:** Clinical summary of contact transmission after introduction of VSNJV by bite of infected black flies to a cattle population

**Experiment / virus**	**Inoculation**	**Animal ID (No. flies feeding)**	**Days of viral shedding (PID)**	**Max viral titer log**_**10**_**TCID**_**50**_**/ml (Location)**	**SN titer**
Experiment 1					
NJ82AZB	Inoculated	528 (6)	4	5.26	>256
		620 (4)	4,7	4.05	>256
	Contact	161	-	-	<4
		282	-	-	<4
		368	-	-	<4
		587	-	-	<4
NJ95COE	Inoculated	001 (9)	4,7	3.05	>256
		579 (4)	-	-	128
	Contact	026	-	-	<4
		034	-	-	<4
		160	-	-	<4
		483	-	-	<4
Experiment 2					
NJ82AZB	Inoculated	034 (4)	1-7	6.10	>256
		368 (11)	1-5	3.6	>256
		587 (3)	1-6	4.39	>256
	Contact	282	-	-	<4
		483	-	-	<4
		026	-	-	<4
		160	8	<2.17 (Tonsil)	64
		161	6	<2.17 (Tonsil)	<4

In experiment 1, swab samples were only collected on PIDs 1, 4, 7, and 9. In experiment 2 swab samples were collected daily.

In the second contact experiment, where only NJ82AZB was examined, peak viral shedding varied among the primary animals (Table 4). Contact animals did not develop clinical signs; however, positive nasal and tonsil swabs were collected on PID 6 from one contact animal, and a positive tonsil swab was collected from a second contact animal on PID 8. Serology of the first animal indicated seroconversion, to a dilution of 1:64, while the second animal remained seronegative.

## Discussion

An in depth discussion of VSNJV ecology in livestock populations has been previously described [24]. Results of the current study and the impacts on VSNJV ecology are included here. The results of the current study, when considered with the results of phylogenetic analysis of VSNJV strains, support the theory of VSNJV host predilection in a cattle model. Clinical disease scores were significantly greater in cattle inoculated with NJ82AZB, NJCOB, and NJWYE, than those infected with a swine strain (NJOSF). Though clinical scores were not significantly different between cattle infected with VSNJV isolated from an outbreak primarily affecting cattle (NJ82AZB and NJ82COB) and those infected with VSNJV isolated during an outbreak primarily affecting horses (NJ06WYE) it is important to note that lameness and discomfort was only observed when experimental animals were infected with a homologous virus strain. When compared to previous results [25], clinical severity and shedding patterns of animals infected with NJ06WYE were more similar to cattle infected by black fly bite with a 1995 VSNJV strain, which was isolated during an outbreak that primarily affected horses.

Animal 566 became infected and presented with clinical disease even though a blood meal was not detected in any of the infected black flies used to inoculate this animal. During inoculation, flies were observed biting the coronary band of this animal though, apparently, a blood meal was not taken. This was also seen in a previous study involving pigs, and indicates that probing by infected flies is sufficient for animal infection [6].

The failure of the positive control in the NJ82AZB trial to become infected following scarification of the lip and coronary band must be addressed. It would be expected that a more virulent strain of virus would cause infection especially since all other positive controls became infected. It is not known why this result was observed, but previous studies have indicated that infection of cattle via the bite of infected black flies is more efficient than scarification [25,26]. This animal was also the smallest of any utilized in the study (175 kg). Disease is usually absent in antibody free cattle less than 1 year old as observed during early outbreaks in the United States [27]. The size of the control animal may indicate an age less than 1 year old and that it is not susceptible to infection.

The results in swine are less conclusive. Extent and duration of viral shedding for both NJ82AZB and NJ06WYE after exposure to infected black flies was comparable to what has been observed in previous studies of swine infected by the same route with a VSNJV strain isolated during the 1997 outbreak, which primarily affected horses. Previous studies demonstrated similar levels of viral shedding in swine after infection with a swine isolate of VSNJV [12,13]. Collectively, these results do not indicate host predilections for VSNJV strains in swine, and are dissimilar to previous results which demonstrated a decreased virulence of NJ82AZB in swine [23]. It must be noted that direct comparisons cannot be made between these studies, since inoculation routes and inoculation doses were different. Application of VSNJV to scarified mucosa or by intradermal injection requires much higher doses of virus to achieve consistent infection than in the black fly inoculation model. Previous studies have inoculated with approximately 10^5^-10^6^ TCID_50_ VSNJV [12,14]. In the current study, saliva collected from infected black flies contained a maximum of 10^2.4^ plaque forming units/ml. Even when considering multiple fly bites, the dose of VSNJV is much lower in the current study, yet extent and duration of virus shedding are comparable or greater. This may indicate that while virus strain does not appear to impact clinical severity, inoculation route might.

The results presented here indicate that infection with different strains of VSNJV result in differences in clinical severity in cattle, but not in swine. The impact of viral strain on clinical severity in other hosts, such as horses, is not known. Clinical severity of VSNJV infection is important because detection of VS in US livestock populations largely depends on observation of clinical disease in infected animals. Clinical disease in livestock can range from severe to unapparent [28]. Viral host predilections, which result in variable disease severity, may result in an underestimation of viral prevalence in livestock populations during outbreaks. Unapparent or mild clinical cases may be missed, resulting in further spread of the virus during an outbreak. Mild or unapparent infections due to viral predilections may contribute to the unequal numbers of investigations and identifications of VSNJV infections in one species over another during epidemics of VSNJV.

Severity of clinical disease has added significance because of its potential impact on transmission of the virus, as well as detection in vertebrate hosts. Transmission of VSNJV in livestock models has recently been investigated, and multiple routes, consistent with vector-borne, mechanical, and contact transmission have been validated [5,6,10-12,14,25,26]. Most routes of VSNJV transmission utilize vesicular fluid or infected saliva of the vertebrate host as the source of inoculum, because viremia is not present in infected livestock [2,5,6,11-14,26,28].

With clinical vertebrate hosts serving as a primary source of virus for transmission of VSNJV, clinical severity and extent and duration of viral shedding should be considered when examining the epidemiology of the virus. Animal-to-animal contact transmission, as well as virus acquisition by insect vectors, relies upon the amount of virus present. It was previously demonstrated that consistent infection via scarification, which mimics contact transmission or infection via contaminated feed or equipment, requires a dose of 10^6^-10^7^ TCID_50_ of virus [29]. Although it has not been examined, it is expected that the efficiency of VSNJV transmission to potential vectors feeding on or near vesicular lesions would increase along with viral concentration. Increases in duration of viral shedding would allow for increased duration of contact among infected and naive animals, as well as a larger time window for vectors to acquire virus from an infected host.

## Conclusions

During an outbreak, host predilection of VSNJV strains could result in more severe clinical disease in one host, over another. In populations of hosts with severe clinical signs, higher rates of detection and virus transmission would be expected. In populations of livestock where clinical signs are unapparent or mild, virus transmission rates may be lower, yet detection could be lower as well, allowing undetected virus spread to occur. In the contact transmission experiments, animal-to-animal contact transmission was observed with NJ82AZB, but not with NJ95COE. Virus titers for animals infected with NJ82AZB were at least one log_10_ higher and present through PID 7, whereas those infected with NJ95COE did not yield positive swab samples after PID 4. Peak titers were observed at PID 5 and 6 for NJ82AZB animals and on PID 1 for NJ95COE animals, suggesting that virus was present at higher concentrations and for a longer period from animals infected with the NJ82AZB. Although contact transmission in cattle was not observed with experimental animals infected with a heterologous VSNJV strain, it cannot be concluded that contact transmission of this virus would not occur among cattle in a natural setting. It is likely that after introduction of a heterologous strain of VSNJV into a population, contact transmission would occur when population densities are high, but at a lower rate than if infected with a homologous strain.

Further work is needed to examine VSNJV strain host predilections in additional hosts. Experimental verification of the trends observed in the current study in other livestock hosts, such as horses, would add to the understanding of VSNJV epidemiology. Additionally, fine scale genetic analysis of isolates obtained during outbreaks may provide additional information that may be used to identify virulence factors associated with VSNJV. This information could further illuminate the ecology of VSNJV during epidemics in livestock. Monitoring and control efforts during epidemics could be modified to better track and isolate VSNJV in livestock populations.

## Methods

### Host predilection

#### Black flies, experimental animals, and VSNJV strains

Laboratory-reared black flies, *Simulium vittatum* Zetterstedt (IS-7 cytotype), were utilized to infect experimental animals. The vesicular stomatitis New Jersey viruses used in this study were isolated from tongue epithelium of naturally infected animals during the 1982, 1995 and 2006 epidemics, and from an infected sand fly collected on Ossabaw Island GA in 1987 (NJOSF). Feral swine are the primary vertebrate host for VSNJV on Ossabaw, and this virus is considered a swine strain of VSNJV. The 1982 viruses were isolated from the tongue epithelium of a cow from Colorado (NJ82COB: NVSL Accession no. 82-31860), and a cow from Arizona (NJ82AZB: NVSL Accession no. 82-25991), the 1995 virus was isolated from the tongue epithelium of a horse from Colorado (NJ95COE: NVSL Accession no. 00778) and the 2006 virus was isolated from tongue epithelium of a horse from Wyoming (NJ06WYE: NVSL Accession no. 452595). Fifty-two mixed breed dairy steer or bull calves (*Bos taurus* L.) weighing 175 to 300 kg, and nine swine weighing 12-22 kg were obtained from experimental animal providers (Cabaniss Cattle Company, Stephens GA, Alan Cagle, Madison GA, Valley Brook Farm, Madison, GA, USA) and acclimated for one week in the Animal Health Research Center, a BSL-3Ag large animal containment facility at the College of Veterinary Medicine, The University of Georgia, prior to initiation of the experiment. The use of animals was approved by the University of Georgia’s Institutional Animal Care and Use Committee (approval no. 2008-08-22).

#### Cattle infections

Prior to the initiation of experiments, female black flies were infected with 10^3.5^ plaque forming units (pfu) of either NJ82COB, NJ82AZB, NJ06WYE, or NJOSF via intrathoracic inoculation and held for three days extrinsic incubation (the time period between acquisition of an infectious agent by a vector and the vector’s ability to transmit the agent to a susceptible vertebrate host) as previously described [5]. For each virus, animals were randomly assigned to one of three treatment groups. Group 1 was comprised of three animals that were designated for infection on the muco-cutaneous junction of the lower lip (lower lip), two animals were in group 2 and were infected on the neck, and the two animals in group 3 were infected on the coronary band. One animal was designated as a positive control and infected on the lower lip and on the rear coronary band via scarification and application of 100 μl of a VSNJV solution with a titer of approximately 10^7^ TCID_50_/ml.

Animals were sedated (0.1-0.3 mg/kg xylazine, IM) and feeding cages containing 30 infected flies were manually held for 20 to 30 min on the designated areas of sedated animals. The designated feeding sites were shaved using commercially available double bladed disposable razors 24 hours prior to black fly feeding. When feeding took place on the muzzle, cages measuring 2.5cm × 1.5cm × 5cm were built from 5cm × 5cm Plexiglas tubing that was cut in half and sealed with plastic cut from a Petri dish on two sides. The remaining opening was covered with polyester mesh (12 squares/cm). A hole was drilled to allow placement of flies into the cage and then sealed with a laboratory cork. Cages used for feeding on the coronary band were designed with a curved feeding surface to allow for better contact with the coronary band. Areas where cages were held on animals were clearly marked with permanent ink for swab sampling. The number of VSNJV-inflected black flies feeding on each host was determined by dissection and visual observation of blood in the midgut.

#### Swine infections

Prior to initiation of experiments, female black flies were infected with either NJ82AZB or NJ06WYE as described. For NJ82AZB, three animals were designated for inoculation on the planum rostrale, two for inoculation on the coronary band, and one on the abdomen. For NJ06WYE, three animals were designated for inoculation on the planum rostrale. Animals were sedated (Telazol 2 mg/kg and xylazine 2 mg/kg, intramuscularly), and feeding cages containing 30 infected female black flies were held on the designated feeding site for 20 to 30 minutes. Feeding cages were constructed from 5-cm-diameter PVC or polycarbonate tubing cut into 1.3-cm sections and enclosed on the two sides with polyester mesh (12 squares/cm). Feeding cages used on the coronary bands were constructed as described previously.

#### Sampling and disease scoring

Experimental animals were observed daily for vesicle formation, and nasal, oral, and bite site swab samples were taken for virus isolation on Vero cells. Swab samples were placed in individual cryotubes containing 1 ml of viral transport medium (Minimum Essential Medium supplemented with 3% fetal bovine serum, 1000 U penicillin G, 1 mg streptomycin, 0.25 mg gentamicin sulfate, 0.5 mg kanamycin monosulfate, and 2.5 μg/ml amphotericin B). Blood was collected daily through jugular puncture for virus isolation and serum neutralization assays.

Disease scores were assigned to each animal based on the following criteria: Lesion Score 0-3 (0=no lesion, 1= lesion < 1.5cm, 2= lesion > 1.5cm, 3= lesion>1.5cm and development of secondary lesion), Shedding score 0-1 (0= no consecutive days of virus shedding, 1=>1 consecutive days of virus shedding), and Lameness score 0-1 (0= no lameness/discomfort, 1= obvious lameness/discomfort). Total clinical scores were determined by adding the three separate scores together (a maximum score of 5 would indicate development of large, visible vesicular lesions, viral shedding for 2 or more days, and exhibition of obvious discomfort/lameness; a low score of 1 or 2 might indicate that a small lesion developed, virus shedding was not detected on consecutive days, and the animal did not appear to be lame or in discomfort). Animals were euthanized on post-infection day (PID) 13-14, and tissues were collected from the inoculation site, secondary lesions, tonsil, and regional lymph nodes. Tissues were processed for virus isolation, histopathology, and immunohistochemistry.

#### Statistical methods

Maximum viral titers and shedding duration were analyzed by an analysis of variance using Stata 10 statistical software. Total disease scores were analyzed by a non-parametric Kruskal-Wallis test of rank, using the same statistical program. Animals inoculated on the neck were not included in the analysis of clinical scores. These animals were excluded from clinical scoring because previous infection studies indicate that inoculation on a haired area such as the neck does not result in clinical presentation of disease. Upon detection of differences among viruses, a pairwise comparison was made utilizing the Dwass-Steel-Critchlow-Fligner test.

### Animal-to-animal contact transmission

In independent trials, animal-to-animal contact transmission following infection with the NJ82AZB and NJ95COE viruses was investigated in cattle. For each virus, 2 primary steers were infected by infected black fly bite as previously described on the lower lip. After 24 hr, these animals were placed in contact with 4 seronegative, naïve steers. Animals were housed in ~56 m^2^ containment rooms. Feed and water was provided for animals in communal feed troughs and water buckets. Animals were observed daily for lesions and oral, nasal, and tonsil samples were collected on PIDs 1, 3, 7, and 9 from primary and contact animals. Blood was collected on the same days from all animals for serum neutralization assays. On PID 11, primary animals were euthanized, and tissues were collected as previously described. Contact animals were monitored through day 15 for serological signs of infection.

A second contact transmission experiment was carried out utilizing the seronegative contact animals from the first contact experiment, with a change in the ratio of primary to contact animals. In this experiment, 3 animals were infected with NJ82AZB via black fly bite and were placed into contact with 5 seronegative animals. Swab and serum samples were collected and processed daily as previously described.

### Virus isolation, titration, and neutralization

Vero Middle America Research Unit (MARU, [Vero M]) cell culture monolayers were used for all virus isolations and titrations. Swab samples were vortexed for approximately 30 sec. All samples were clarified by centrifugation (10,000 rpm for 10 min) and 100 μl of the resulting supernatant were transferred to individual Vero M cell monolayers in 24 well plates and observed for cytopathic effects. Titration of virus isolation-positive samples was performed via end-point titration, and reported as TCID_50_/ml of transport medium. All isolates were confirmed as VSNJV utilizing reverse transcription PCR with VSNJV specific primers [30].

Sera collected for neutralization assays were heat inactivated at 56°C for 30 min. Two-fold serial dilutions (starting from 1:4) of the serum were incubated with approximately 10^3^ TCID_50_ doses of the same VSNJV strain used for infection of experimental animals for 1 h at 37°C in 96-well microtitre plates (25μl serum:25μl virus solution). Following inculation, 150μl of Vero cell suspension containing 300,000/ml was added to each well containing serum/virus mixture. Plates were covered with loosely fitting lids and incubated for 72 hours at 37°C in an atmosphere of 5% CO_2_. Wells were observed for cytopathic effects. Cell control, virus control, positive control, and negative control serum were included in each neutralization assay. Samples were considered positive if cytopathic effects were absent.

## Competing interests

The authors declare that they have no competing interests.

## Authors’ contribution

PFS participated in study design, participated in all experiments, assisted with analysis of results, and drafted the manuscript. EWH and DC participated in all experiments and provided pathology expertise throughout the course of the described work. EWG and RN reared black flies and provided flies for animal infections. RB participated in results analysis. DES participated in study conception and participated in study design. DGM participated in study conception, participated in design and coordination, and participated in all experiments. All authors read and approved the final manuscript.
